# Multiscale Traffic Sign Detection Method in Complex Environment Based on YOLOv4

**DOI:** 10.1155/2022/5297605

**Published:** 2022-10-22

**Authors:** Yongjie Wang, Miaoyuan Bai, Mingzhi Wang, Fengfeng Zhao, Jifeng Guo

**Affiliations:** College of Information and Computer Engineering, Northeast Forestry University, Harbin 150040, China

## Abstract

Traffic sign detection is a challenging problem in the field of unmanned driving, particularly important in complex environments. We propose a method, based on the improved You only look once (YOLO) v4, to detect and recognize multiscale traffic signs in complex environments. This method employs an image preprocessing module that can classify and denoize images of complex environments and then input the images into the improved YOLOv4. We also design an improved feature pyramid structure to replace the original feature pyramid of YOLOv4. This structure uses an adaptive feature fusion module and a multiscale feature transfer mechanism to reduce putative information loss in the feature map generation process and improve the information transfer between deep and shallow features, enhancing the representation ability of feature pyramids. Finally, we use EIOU LOSS and Cluster-NMS to further improve the model performance. The experimental results on the fusion of Tsinghua-Tencent 100 K and our collected dataset show that the proposed method achieves an mAP of 81.78%. Compared to existing methods, our method demonstrates its superiority with regard to traffic sign detection.

## 1. Introduction

Automatic traffic sign detection and recognition (ATSDR) is a topic attracting immense interest in the computer vision field. It plays a very important role in the advanced auto drive system. Due to the diversity of traffic sign types and the complexity of actual road conditions, a real-time and high-precision solution of ATSDR remains a challenging problem [1]. Vehicles will inevitably encounter various extreme environments (i.e., rain and snow, fog, and blurred vision caused by other reasons), which significantly increases the difficulty of traffic sign detection and recognition [2]. ATSDR can be divided into two parts, traffic sign detection (TSD) and traffic sign recognition (TSR), processed consecutively. The detection step is used to locate the areas containing traffic signs in the image, and the recognition step is used to classify these areas into specific traffic signs or backgrounds.

The traditional TSD method emphasizes the use of color features or shape features of traffic signs for detection. Detection methods based on color features usually detect bright traffic signs, which discern the surrounding environment well [3, 4]. However, the detection method based on color features is easily affected by the external environment; especially in extreme weather, the efficiency of this detection method is significantly reduced [5]. The detection method based on shape features works by first detecting the shape contour first, and then making a decision according to the number of contours. However, in a complex environment, traffic signs are easily blocked by other objects, which seriously affects the detection efficiency. With the development of deep learning, traffic sign detection and recognition algorithms based on the convolutional neural network have been widely studied. These algorithms can automatically locate and identify traffic signs, significantly improving the recognition speed [6]. However, traffic sign detection and recognition still face the following challenges:

1. In rainy, snowy, foggy, and other complicated weather, the photos captured by the camera contain a significant amount of noise [7]
2. Under different lighting conditions, the color and saturation of traffic signs will change [8]
3. Some part of the traffic sign may be blocked by railings, trees, snow, etc.
4. Under different shooting angles, the shape of traffic signs may be distorted
5. Some traffic signs are too small to be recognized

You only look once, (YOLO) [9] as a new object detection model, which not only achieves fast detection but also high accuracy. In recent years, the YOLO model has been continuously improved, with steady enhancements in its performance. YOLO v2 adds batch normalization to YOLO to further improve detection accuracy [10]. YOLOv3 deeply refines the feature extraction network and adopts a multi-scale fusion method for prediction, which effectively improves the detection accuracy while maintaining a high detection speed [11]. YOLOv4 combines the cross-stage partial network in the trunk part and introduces the spatial pyramid pool and path aggregation network in the neck part, which reaches a new standard in the detection accuracy and speed [12]. However, under extreme environmental conditions, the accuracy of YOLOv4 is not ideal, and there is still room for improvement. Therefore, we propose a multiscale traffic sign detection method based on YOLOv4 in complex environments. Experimental results on the fusion of Tsinghua-Tencent 100 K (TT-100K) and the data set collected by us show that the detection accuracy of this method is significantly improved, and the detection speed is guaranteed.

The main contributions of this study are as follows:

1. We propose an image preprocessing module for addressing high noise in complex environments. This module classifies noise and uses corresponding preprocessing algorithms to reduce the impact of noise on traffic sign recognition.
2. We propose an improved feature pyramid structure (FT-Feature pyramid networks,FT-FPN). The structure includes an adaptive feature fusion module (AFFM) and a multiscale feature transfer mechanism (MFTM). Through these two parts, more information is retained during the feature transfer, which enhances the expressive ability of the feature pyramid and effectively improves the accuracy of multiscale target recognition.
3. EIOU loss is used to replace the CIOU loss employed by YOLOv4 to optimize the training model, improve the speed and accuracy of the algorithm, and use the weighted Cluster-NMS to replace the DIOU-NMS algorithm of YOLOv4 to improve the accuracy of generating detection frames.

Section 2 of this paper reviews the general object detection framework and the traffic sign detection method based on convolutional neural network (CNN).  Section 3 introduces our improved ideas and describes in detail the specific methods of our proposed model; Section 4 compares, analyzes, and evaluates our method with other existing methods; Section 5 presents the summary and prospects of future work.

## 2. Related Work

### 2.1. Object Detection

Object detection, among the core problems in the field of computer vision, involves detecting the objects of interest in the image and determining their categories and positions. Because of the different appearance, shape, and posture of various objects, as well as the interference of illumination and occlusion, object detection is consistently the most challenging problem in the field of computer vision. Zou et al. [13] reviewed more than 400 papers on the development of object detection technology, including historical milestone detectors, detection frameworks, evaluation indicators, data sets, acceleration techniques, and detection applications systematically and comprehensively presenting the development status of object detection.

The research process of the object detection algorithm can be roughly divided into two stages, namely, the conventional method stage and the deep learning based method stage. The traditional object detection method comprises three steps: feature extraction, region extraction, and classification regression. Traditional object detection methods rely on sliding windows and manual feature extraction. The histogram of oriented gradient proposed by Dalal and Triggs [14] is calculated on a dense grid of uniformly spaced cells, and overlapping local contrast normalization is used to improve performance. Due to the limited ability of manual feature extraction, it cannot meet the needs of object detection. Therefore, object detection algorithms based on deep learning technology have undergone rapid development. At present, the mainstream deep learning based methods can be roughly divided into two categories: the two-stage method and the one-stage method. The two-stage method divides the problem into two parts. First, the location information of the object is determined, and then the objects in the region of interest are classified and regression is employed. In the last five to seven years, around 367 papers showcased an architectural change in CNN [15]. RCNN [16] is the first algorithm that successfully applies deep learning technology to object detection. It employs CNN to extract object features and employs selective search to reduce the amount of computation in the regional suggestion stage. A series of improvements have been made to the original RCNN algorithm, such as Fast RCNN [17], and Faster-RCNN [18]. The one-step method transforms the object detection task into a regression problem, which can directly generate the classification and position coordinates of the object. Typical methods include YOLO, SSD [19], and RetinaNet [20]. Generally, the two-stage method is more accurate, but the one-stage method exhibits better detection speed and its accuracy is constantly improving. As time went by, some algorithms have been improved to achieve specific tasks. Pang et al. [21, 22] proposed an unsupervised cross-domain ReID method based on median stable clustering (MSC) and global distance classification (GDC) to improve the performance of cross-domain person reidentification (ReID). Patel et al. [23] proposed the Dimension-Based Generic Convolution Block (DBGC), which can be used with any CNN to make the architecture generic and provide a dimensionwise selection of various height, width, and depth kernels.

### 2.2. TSD Based on CNN

The CNN has been widely used in computer vision, natural language processing, and other fields in recent years owing to its powerful feature extraction capability [24, 25]. Researchers have improved the traditional object detection algorithm to improve the accuracy of TSR. Shao et al. [26] proposed an improved fast RCNN traffic sign detection method. They simplified the Gabor wavelet with a region suggestion algorithm to improve the network recognition speed. Wang et al. [27] used the RFP structure to replace the original SPP structure and added attention mechanisms CBAM and CA structures to the backbone and neck layers of the model, which ultimately reduced the parameters of the model and improved the inference speed. Wu and Liao [28] improved the SSD, used RFM to improve the receptive field and semantics of the predicted feature map, and introduced a path aggregation network to fuse multi-scale features to improve the location and classification accuracy of traffic signs. Yang and Bingfeng [29] proposed a lightweight real-time traffic sign detection integration framework based on YOLO by combining deep learning methods. The framework optimized the latency problem by reducing the computational overhead of the network and facilitated the transmission and sharing of information at different levels.

Currently, most networks use a single-scale depth feature, which is difficult to obtain in a complex environment, and its accuracy is not ideal. In complex environments, feature extraction of traffic signs is susceptible to various noise types, and the proportion of traffic signs in the whole image is very limited. Therefore, multiscale feature extraction is particularly important in TSR [30]. FPNs are the basic component of the recognition system for detecting objects of different scales. FPN improves model accuracy by extracting multi-scale feature information for fusion. However, due to the reduction of feature channels, a large amount of information will be lost for advanced features, leading to a decrease in the detection accuracy [31]. To deal with this problem, researchers proposed a receptive field pyramid (RFP) [32], which can enhance the expressive ability of FPN and enable the network to learn the optimal feature fusion mode.

## 3. Proposed Method

Traffic sign detection and recognition is very important for automatic driving, especially in extreme environments. In the real scene, the environment is complex and changeable, such that the image captured by the vehicle camera may contain a significant amount of noise, which has a serious impact on the detection and recognition of traffic signs. At present, the recognition performance of mainstream models in complex environments is not satisfactory, and only one scene can be recognized. To improve the recognition rate and robustness of the model in a complex environment, we improved YOLOv4. This section describes the Classify Denoizer module, Feature Pyramid Networks, EIOU loss, and cluster-NMS in detail.

### 3.1. Classify Denoizer Module

In the task of TSD in a complex environment, the image in the dataset contains evident noise due to various reasons. Rain, snow, fog, and inevitable image blurring under complicated weather pose great challenges to the detection of traffic signs. To solve the problems of low image quality and evident complex noise, we propose a preprocessing method for TSD tasks in complex environments. Before the images are input into the detection model, they are added into the Classify Denoizer module for preprocessing, after which they are input into the detection model.

The proposed Classify Denoizer module mainly consists of the Challenge Classifier and Denoizing block. The Challenge Classifier is based on the VGG16 [33] network model for feature extraction and classification of the original image. We train and adjust the VGG16 network through transfer learning and fine-tuning methods, such that it can achieve the classification of the input image. The Challenge Classifier trained by us can classify the original dataset into five types, and the classified dataset will be used as input of the Denoizing Block for corresponding denoizing processing.

The Denoizing Block includes four parts as removal algorithms for rain, snow, fog, and blur. The rain removal algorithm can reduce the noise generated by raindrops in the image. The snow removal algorithm removes most of the snow, stripe, and veil effects (similar to fog or mist). At the same time, the haze removal algorithm can make up for the missing feature information from high-resolution features while removing the noise in the image and improving the image quality. In addition, the deblurring algorithm solves the problem of image blurring caused by vehicle turbulence. The Denoizing Block removes the evident noise in the image to the maximum extent, changing the image under the complex environment into the normal environment, and restoring the image under the complex background as much as possible. The proposed Denoizing Block combines the currently best-performing denoizing algorithm and continues specific processing to the traffic sign, which optimizes the denoizing effect of the algorithm.

**Figure 1:**
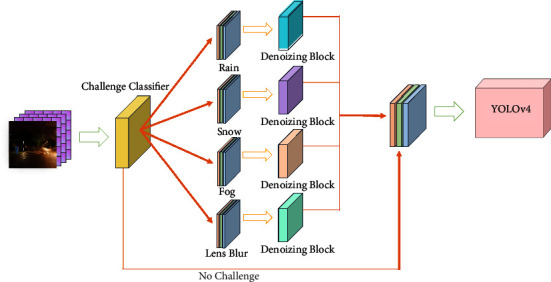
Structure of Classify Denoizer module.

The detection technology of traffic signs in a normal environment is extensively developed. The proposed Classify Denoizer module restores the image in a complex environment to the greatest possible extent to the image in a normal environment through preprocessing technology and detect traffic signs, which significantly improves the model performance. The proposed Classify Denoizer module is illustrated in Figure 1.

Figure 1 shows the main process of the Classify Denoizer module. The image processing of this module is divided into four steps:

1. First, original images are input into the trained Challenge Classifier, which detects original input according to the noise type in the image
2. If noise is detected, it is divided into four challenge types according to our settings: rain, snow, fog, and lens blur. The classified images are used as the input for different denoizing blocks
3. Different Denoizing Blocks denoize the input images, and finally the processed images are fed into the improved YOLOv4 model
4. If the detected challenge type is “no challenge”, the Challenge Classifier accepts it as a normal image and directly transmits the image to the improved YOLOv4 by skipping the Denoizing Block

#### 3.1.1. Challenge Classifier

For the Classify Denoizer module, these challenging images must be classified before they can be correctly entered into the corresponding denoizing algorithm. If the image is incorrectly classified, the subsequent detection performance will be reduced. Therefore, accurate classification is of high importance for the Classify Denoizer module. The VGG16 network model exhibits excellent classification performance. The model has a simple structure and numerous structures adopt the same parameters. Simultaneously, the model is also composed of several convolutional layers and pooling layers in the way of stacking, which easily forms a deep network structure.

We adopt the VGG16 as the basic structure of our Challenge Classifier and introduce transfer learning and pretraining on an ImageNet dataset to obtain model parameters that can recognize low-level features of images. As the resolution of the feature image decreases, the number of model channels will increase exponentially, so as to retain the semantic information of the image to the greatest possible extent, and gradually combine the texture features of the image into category features. VGG16 includes two stages, namely, feature extraction and classification. The feature extractor compresses existing information in the image into a low-dimensional feature space. Subsequent phases use these characteristics to perform the desired classification.

- Feature extractor: the feature extractor is composed of 13 convolution blocks, each of which has a 3 × 3 convolution kernel, a batch normalization layer, and a ReLU activation layer. We use the max-pooling operation for subsampling and perform global average pooling in the final stage to further compares the features.
- Feature classification: loading the VGG16 network model, the underlying model uses ImageNet trained feature extraction layer parameters to apriori fine-tune the top-level network, and the SoftMax activation function to output five kinds of labels, including four different types of noise label (rain, snow, fog, and lens blur) and one “no noise” label.

#### 3.1.2. Denoizing Block

We use the Denoizing Blocks for rain, snow, fog, and blur to deal with the corresponding noise. These Denoizing Blocks are independent of each other, which allows us to add more different noise reduction blocks to improve our model.

Due to the influence of various factors, removing rain is a highly complicated problem. In rainy weather, the details of the background image are covered or lost, resulting in the degradation of image quality. By analyzing and processing the image with the rain removal algorithm, the rain stripe is removed, and the clean background scene is restored, which is helpful to improve image quality and restore image features. Jiang [34] explored multiscale collaboration of rain patterns in a unified framework from the perspective of input image scale and hierarchical depth features, and at the same time carried out a recursive calculation for similar rain strips at different positions to capture global texture. This algorithm provides inspiration for our rain removal module. We improved the algorithm accordingly. Because the denoizing intensity of the algorithm is excessive in its processing of rainy day images, and the traffic signs in the image are often small, the shape of the traffic signs may change after the denoizing algorithm, particularly the edge parts of the traffic signs. Therefore, we reduce the noise reduction intensity of the algorithm. We found that the smaller intensity did not reduce the accuracy of the model, but ensured the quality of the input image. Furthermore, we adjust the algorithm such that it can take the output of the YOLOv4 as the input to the Denoizing Block, which smoothly outputs the image into YOLOv4.

Snow days are similar to rainy days, but manifest like a combination of rainy and foggy days, and the research on desnowing algorithms is incomplete. The single image desnowing algorithm using hierarchical dual-tree complex wavelet representation and contradict channel loss [35] is one of the currently best algorithms. The algorithm is mainly aimed at the effect of snow, snow strip, and veil in the image and can deal with scattered noise very well. In TSD, the shielding of large snowflakes on traffic signs will severely affect the detection performance of the model and reduce the recognition accuracy. This algorithm is evidently superior to other snow removal algorithms in terms of the snow removal effect and computational complexity. This also enables our classification noise reduction module to spend less time dealing with the noise in complex environments.

The existence of a large number of small droplets, colloids, dust, and other particles in the air, these particles become suspended in the atmosphere and lead to the formation of fog. Foggy days greatly reduce the quality of a captured image, which is reflected in its reduced contrast and saturation. This makes it difficult to distinguish the contours of the target, thus affecting the detection and recognition of traffic signs. To solve such problems, we use a multiscale enhanced fog removal network [36] based on U-Net. The network steps back to a fog-free image by adding strengthen-operate-subtract to the decoder. The extensive evaluation shows that this model performs best on benchmark datasets.

In the process of vehicle driving, due to the uneven road surface, the on-board camera may have a fuzzy problem when capturing the image. Simultaneously, due to the excessive speed, the local area of the image may also have a fuzzy problem. These ambiguities also reduce the detection performance of traffic signs. We use self-supervised meta-auxiliary learning to improve the performance of deblurring by integrating both external and internal learning. To further optimize the pretraining model, we refer to a novel meta-auxiliary training scheme [37]. This scheme is of great help to our deblurring block.

**Figure 2:**
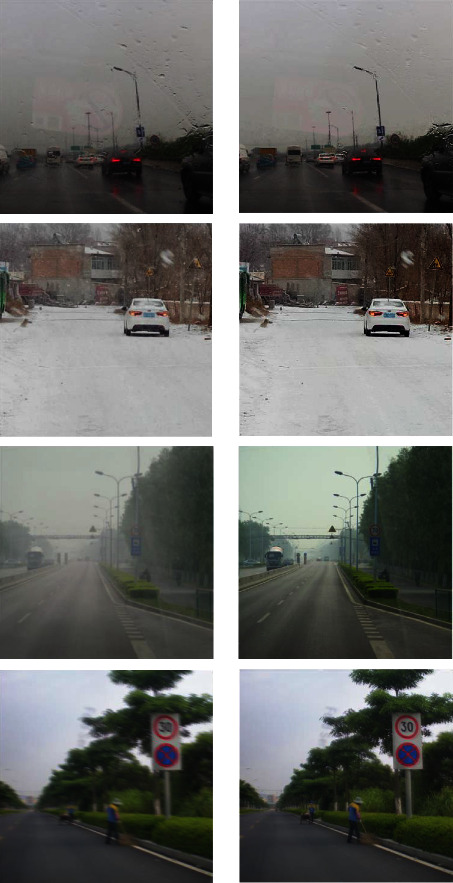
Image processed by the Classify Denoizer module.

Through the Classify Denoizer module, we can efficiently reduce the noise in the image and its impact on the identification of traffic signs.  Figure 2 shows the image processing effect of the Classify Denoizer module.

### 3.2. FT-FPN Structure

**Figure 3:**
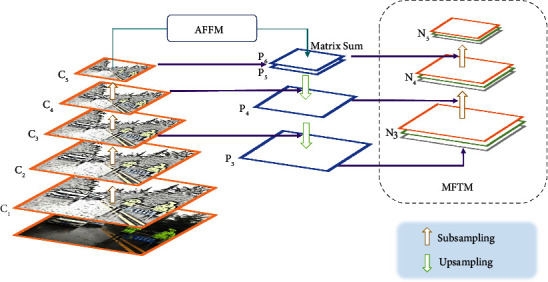
FT-FPN structure.

The characteristic interaction in the original YOLOv4 network is a propagation structure that combines top-down and bottom-up approaches. Although the increase of fast connections shorts the information path between shallow and deep features and speeds up information fusion, the efficiency of nonadjacent information transmission between deep and shallow features remains limited. Furthermore, the traditional feature pyramid network will lose the context information in the high-level feature graph due to the reduction of feature channels. The scale of traffic signs in the image is different, and the influence of noise in the complex environment makes it more difficult to extract the features of traffic signs. To solve the above-given problems, further optimize the interaction between deep and shallow feature information and improve the target detection accuracy, an AFFM and a multiscale feature transfer mechanism are proposed. The FT-FPN structure is shown in Figure 3.

#### 3.2.1. Adaptive Feature Fusion Module

AFFM can be divided into two steps. First, we use the adaptive averaging pool layer to obtain multiple context features at different scales. We set the pooling coefficient as [0.1, 0.5] and adaptively change the target size of the dataset. Then, the spatial attention mechanism generates spatial weight maps for each feature map. The spatial weight map can fuse context features and generate a novel feature map containing context features. The new feature map combines the original high-level feature map. Finally, the combined feature map is fused with other low-level features.

**Figure 4:**
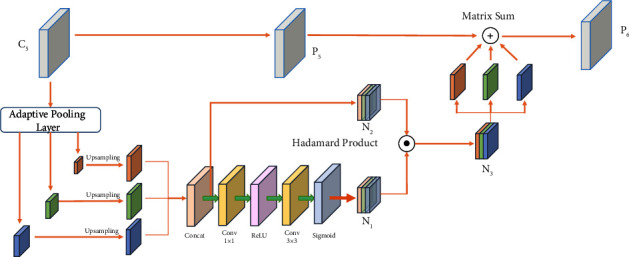
Structure of adaptive feature fusion.

The structure of AFFM is shown in Figure 4. Herein, C_5_ serves as the input of AFFM, and its size is S. C_5_ enters the adaptive pooling layer first, and the context features of different scales (*β*_1_ × S, *β*_2_ × S, *β*_3_ × S) can be obtained after its processing. We process each context feature with 1 × 1 convolution to obtain the same 256-dimensional channel. Then, bilinear interpolation was used to upsample the features to obtain S-scale features and perform the subsequent fusion. The concat layer was used to merge the channels of these context features, and the feature maps were successively passed through the following three layers, the 1 × 1 convolution layer, ReLU activation layer, and 3 × 3 convolution layer. At this point, each feature map will generate the corresponding spatial weight map N_1_. The new feature map N_3_ is obtained by the Hadmard product operation between N_1_ and feature map N_2_ after passing through the concat layer. Finally, the feature map P_6_ was obtained by the Matrix Sum operation of separated feature map N_3_ and original feature map P_5_ of C_5_. At this point, the multiscale information in the feature map is preserved, and the information loss caused by the reduction of channels is alleviated.

#### 3.2.2. Multiscale Feature Transfer Mechanism

In traditional FPN, the feature map is obtained by upsampling high-level features and mutual fusion of low-level features, while the path aggregation network further improves the effect of feature fusion by bidirectional feature propagation. To further optimize the integration between the deep and shallow features and transmission strategy, reduce the redundant structure, and improve the model accuracy and robustness, especially for complex environment features, we proposed a novel feature transfer mechanism, i.e., an MFTM. The feature transfer proposed by us is different from the traditional layer-by-layer transfer. This transfer mechanism enables shallow and deep features to be effectively shared and fused. At this time, shallow features are no longer differentiated only for simple objects, and deep features are no longer differentiated only for complex objects. Compared with the traditional feature transfer mechanism, our method enhances the features at all levels from space to semantics more effectively and provides more comprehensive multidimensional information after the fusion of deep and shallow features. This mechanism learns and perceives the rich details and location information of the target by transmitting and sharing different scales of the receptive field content, thereby obtaining clearer and more accurate features.

**Figure 5:**
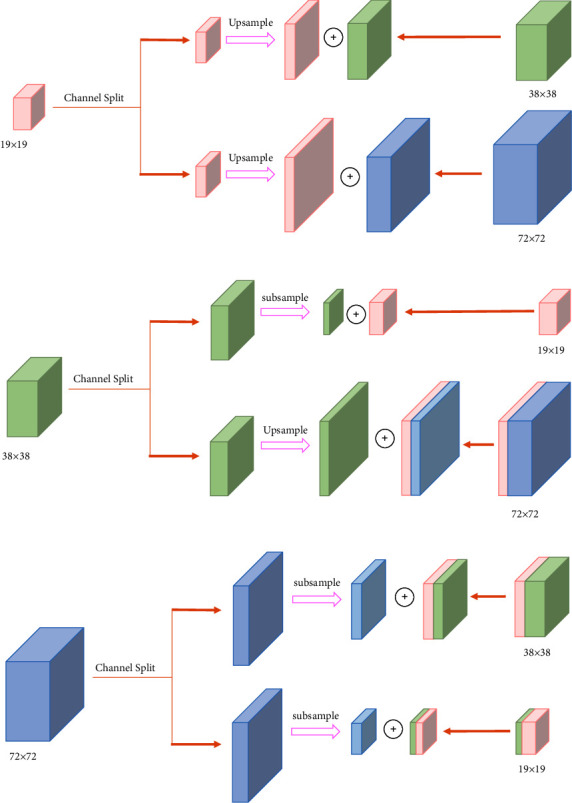
Structure of multiscale feature transfer.

In our mechanism, the features are segmented at each scale, and upsampling or max-pooling operations are applied to assimilate the feature scales. At this point, the generated intermediate features can be shared with each other. Finally, the original and intermediate features of the same scale are fused to obtain multiscale features. MFTM divides feature maps into small-scale features (19 × 19), medium-scale features (38 × 38), and large-scale features (76 × 76). These features are repeatedly fused and share information after upsampling or max-pooling operations, which effectively solves the problem of lack of scale between different layers. The information sharing ability between deep and shallow features is improved, and the detection accuracy of objects is enhanced. The structure of the MFTM is shown in Figure 5.

Figure 5 illustrates the process of MFTM:

1. The 19 × 19 small-scale feature is divided into two parts in the channel dimension, and the upsampling operation is performed on them. Part of them is subjected to ordinary upsampling to obtain 38 × 38 mesoscale features and then fused with the original 38 × 38 mesoscale features to obtain 38 × 38 intermediate features. The other part is subjected to high-power subsampling to obtain 76 × 76 large-scale features and then fused with the original 76 × 76 large-scale features to obtain 76 × 76 intermediate features.
2. The 38 × 38 mesoscale features are divided into two parts in the channel dimension, and the upsampling or subsampling operation is performed on them. Part of them is upsampled to obtain 76 × 76 large-scale features and then fused with the 76 × 76 intermediate features generated in (1) to yield 76 × 76 large-scale feature blocks. The other part is subsampled to obtain 19 × 19 small-scale features and then fused with the original 19 × 19 small-scale features to yield 19 × 19 intermediate features.
3. The 76 × 76 large-scale feature is divided into two parts in the channel dimension, and the down-sampling operation is performed on them. Part of them is subjected to ordinary subsampling to obtain 38 × 38 mesoscale features and then fused with the 38 × 38 intermediate features generated in (1) to yield 38 × 38 mesoscale feature blocks. The other part is subjected to high-power subsampling to obtain 19 × 19 small-scale features and then fused with the 19 × 19 intermediate features generated in (2) to yield 19 × 19 small-scale feature blocks.

At this time, the features of each scale are transferred to the features of other scales, and more information is generated.

### 3.3. EIOU Loss and Cluster-NMS

CIOU loss is used as the loss function of the bounding box, and the CIOU loss function is given by the following formula:

(1) $$\begin{array}{c} L_{CIOU}=1-IOU+\frac{\rho^{2}\left(b,b^{gt}\right)}{c^{2}}+\alpha V\text{,} \end{array}$$

where *b* and *b*^*gt*^ are the center points of the predicted box and the real box, respectively, *ρ* is the Euclidean distance between the two center points, and *c* represents the diagonal distance of the smallest closure area that can contain both the predicted and the real box. *α* is a positive trade-off parameter, V is a parameter used to measure the consistency of aspect ratio, *α* and V are defined as shown in the following formulas:

(2) $$\begin{array}{c} \alpha =\frac{V}{1-IOU+V}\text{,} \end{array}$$

(3) $$\begin{array}{c} V=\frac{4}{\pi^{2}}{\left(\arctan\frac{\omega^{gt}}{h^{gt}}-\arctan\frac{\omega }{h}\right)}^{2}\text{,} \end{array}$$

where *ω*, *ω*^*gt*^, *h*, *h*^*gt*^ are the widths and lengths of the predicted box and the ground truth box, respectively.

Although CIOU loss considers the overlapping area, center point distance, and aspect ratio of bounding box regression, the difference in the aspect ratio is reflected by V in its formula, rather than the true difference between the width and height. This can sometimes affect the performance of the model. To solve this problem, we use EIOU loss to replace the original CIOU loss. The EIOU loss splits the aspect ratio on the basis of CIOU loss and calculates the difference between the width and height. The loss function consists of three parts: the overlap loss, center distance loss, width, and height loss. The first two parts continue the method in CIOU, but the width and height loss directly minimize the difference between the width and height of the target box and the anchor box, making the convergence speed faster. The EIOU loss function is represented by the following formula:

(4) $$\begin{array}{c} L_{EIOU}=L_{IOU}+L_{dis}+L_{asp}=1-IOU+\frac{\rho^{2}\left(b,b^{gt}\right)}{c^{2}}+\frac{\rho^{2}\left(\omega ,\omega^{gt}\right)}{c_{\omega }^{2}}+\frac{\rho^{2}\left(h,h^{gt}\right)}{c_{h}^{2}}\text{,} \end{array}$$

where *c*_*ω*_ and *c*_*h*_ are the width and length of the minimum circumscribed rectangle covering the two boxes, respectively.

Nonmaximum suppression is used to find locally optimal object bounding boxes and eliminate redundant ones. YOLOv4 adopts the DIOU-NMS algorithm, which not only considers the overlapping area but also the center point distance. However, the algorithm still causes false suppression when faced with two very close targets. To improve the accuracy of finding bounding boxes and increase the detection speed, we employ the Cluster-NMS algorithm. The Cluster-NMS algorithm uses row transformation to simplify the iterations that should be performed on all Clusters to iterate only on the Cluster with the largest number of boxes. This significantly reduces the number of iterations and reduces the time complexity. In the detected image, the effect is more evident when there are multiple Clusters. Furthermore, the Cluster-NMS algorithm can fuse the score penalty, weighted average, and center point distance methods to further improve the accuracy. In this study, the Cluster-NMS algorithm was fused with the weighted average method is used to replace the DIOU-NMS algorithm in YOLOv4 to obtain a further increase in the inference speed and accuracy of the model.

## 4. Experiments and Analysis

### 4.1. Datasets and Data Augmentation

Traffic sign datasets play an important role in traffic sign detection and recognition, and the quality of the datasets affects the overall detection results. TT-100K has richer image resources and more pictures in complex scenes. The TT-100K dataset contains 100,000 images, of which 30,000 images contain traffic signs.

**Figure 6:**
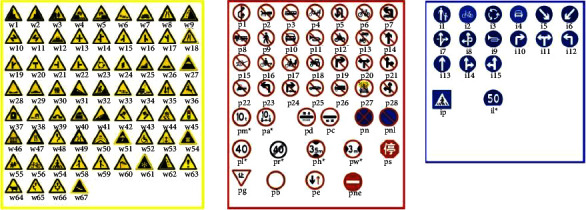
Traffic sign categories in Tsinghua-Tencent 100K.

The brightness and weather conditions of these images are variable. The images in TT-100K are from the Tencent Street View, which covers more than 300 Chinese cities and their road networks [38]. These images are divided into 220 classes, and each class has a unique name (partial classification is shown in Figure 6), which will help better distinguish traffic signs. In the TT-100K dataset, although the number of images reaches 100,000, only about 10,000 images contain traffic signs, and the remaining 90,000 images do not. To expand the size of the dataset we used for recognition and avoid the impact on the recognition rate of our model due to the small data size, we took and collected another 3000 images to expand the dataset. To better train our model and improve its learning ability in complex environments, most images we captured are of rainy, snowy, foggy, and other complex weather. We use Labelme to annotate these images.

**Figure 7:**
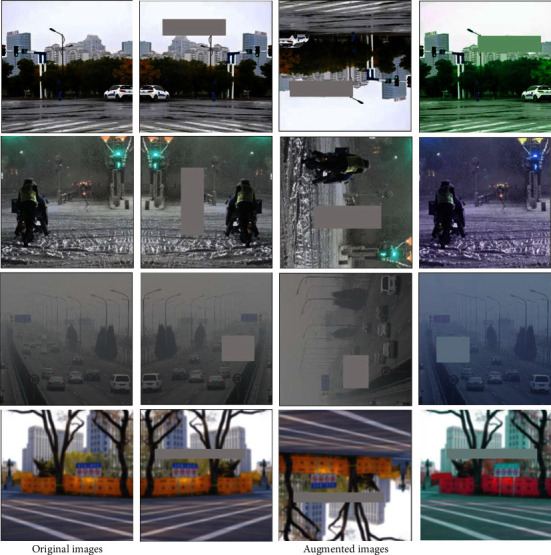
Various sample images after data enhancement.

We obtained 10,000 images with traffic signs in Tsinghua-Tencent 100K, of the 3,000 images captured. Nevertheless, due to the large number of learnable parameters in the model, to prevent the model from overfitting, we used data augmentation methods to expand the dataset. We used the random erasing algorithm [39] to process our original 13,000 images. We also performed operations such as horizontal and vertical flipping, random rotation, and random color transformation on these images. The final result is 39,000 images containing traffic signs. Some images after data enhancement are shown in Figure 7.

### 4.2. Experimental Environment and Evaluation Metrics

This experiment is executed by Python based on the Linux platform, and it is debugged and run on the Ubuntu18.04 server with E5-2680 v4 @ 2.40 GHz CPU and NVIDIA Tesla V100 32G GPU. The training process of the model proposed in this study is implemented based on the Pytorch framework.

The images in the TT-100K dataset have a resolution of 2048 × 2048, and using images of such a large size in the noise classification stage is an expensive process. The noise is distributed over the whole image, and the main function of our Challenge Classifier is to identify the type of noise. Therefore, we enhance this global feature by subsampling and resize the image to 608 × 608 pixels. First, we use a Challenge Classifier to divide the input image into five classes, where four classes are used to represent different noise types, and one class is used to represent no noise (low noise). The Challenge Classifier uses categorical cross-entropy as the loss function, and the Adam optimizer [40] is used to optimize our network during training. The initial learning rate was set to 0.001, and if the validation score did not improve within three epochs, the learning rate was decreased by a factor of 0.5. Finally, we trained our network for 50 epochs using these specifications. The improved YOLOv4 model employs Adam as an optimizer to enhance our model during training. The initial learning rate is set to 0.001, the batch_size is set to 64, and each training is performed for 600 epochs.

To verify the effectiveness and stability of our method for TSD in complex environments, we conduct several sets of experiments to validate and evaluate the performance of the model. We use mean average precision (mAP) and frames per second (FPS) to evaluate the precision and real-time performance of the model and compare and analyze the results with other mainstream models.

In terms of precision, the average precision (AP) is the precision used to measure a specific type of target, and it represents the area under the precision-recall curve. The mean average precision (mAP) is usually used as an indicator to quantitatively evaluate the overall accuracy of the detection model. This is the result of averaging APs of different categories, and it is one of the commonly used indicators for evaluating object detectors. mAP is defined as the following formula:

(5) $$\begin{array}{c} mAP=\frac{1}{N}\overset{N}{\underset{i=1}{\sum }}{AP}_{i}\text{.} \end{array}$$

In terms of real-time performance, we use FPS to evaluate the detection speed of our model. FPS mainly refers to the number of frames per second transmitted by the image. The higher the value, the smoother the displayed action.

### 4.3. Visualization of Detection Results

To more intuitively observe the performance of our model in practical application scenarios, we display the results in a visual form. In this paper, several complex and representative detection results are selected from the experimental results of real road environment scenes.

**Figure 8:**
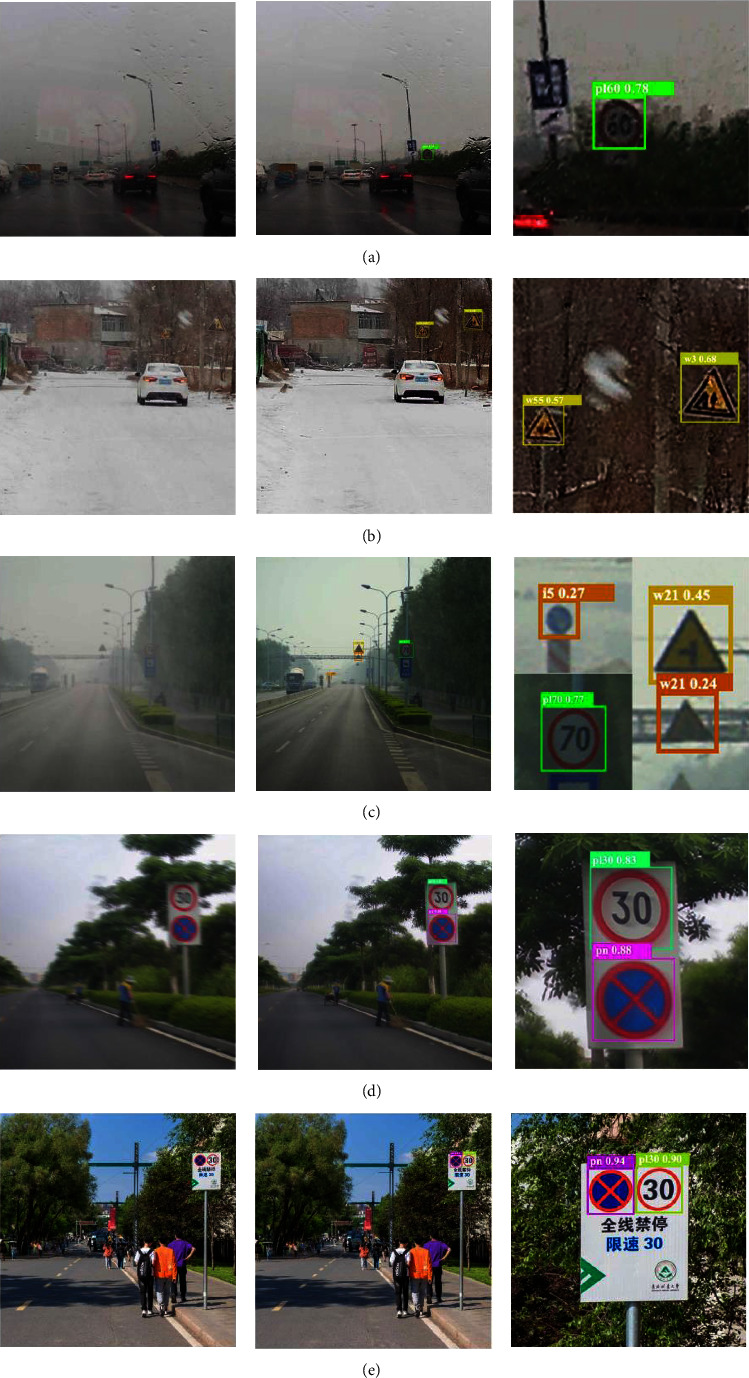
Traffic sign visualization detection results in (a) rain, (b) snow, (c) fog, (d) lens blur, and (e) no challenge environments.

Figure 8 shows representative experimental results in rainy, snowy, foggy, lens blurring, and normal weather. Among them, Figures 8(a) and 8(b) show the detection results in rainy and snowy conditions, respectively. Our model demonstrates the full capability to perform the corresponding detection tasks in rainy and snowy weather and can accurately detect traffic signs. Second, foggy weather is also an important factor affecting the performance of traffic sign detection. The detection results of our model on traffic signs in foggy weather are shown in Figure 8(c). The effect is evident, and it is also very accurate for small-scale TSR. Furthermore, the vehicle will inevitably encounter bumps during the process of driving, which will cause the captured image to become blurred.  Figure 8(d) shows that the model performs very accurately in the detection of traffic signs in the blurred image as well, and the classification results are likewise very accurate. Moreover, we tested the traffic sign detection performance in the normal environment. From the detection effect shown in Figure 8(e), we see that the detection performance in the normal environment is likewise very satisfactory.

The improved model detection results show that in the detection of traffic signs, the location of the bounding box as well as the classification of the traffic signs is highly accurate. These results demonstrate that the detection model proposed in this study not only exhibits reliable accuracy but also meets the detection requirements in complex environments, reflecting good robustness and adaptability of the model.

### 4.4. Performance Comparison

To objectively evaluate the detection performance of our proposed model in real scenarios, this experiment adopts a variety of evaluation indicators from different perspectives for quantitative comprehensive evaluation. Herein, we first analyze the performance of the Challenge Classifier and compare our proposed model with the current mainstream models Faster-RCNN, RetinaNet, YOLOv3, YOLOv4, and SSD. In addition, we also compare the detection performance of the model for traffic signs in different environments. Finally, to more intuitively observe the impact of each of our improvements on model performance, we performed ablation studies on the same dataset and hardware.

#### 4.4.1. Quantitative Analysis

**Figure 9:**
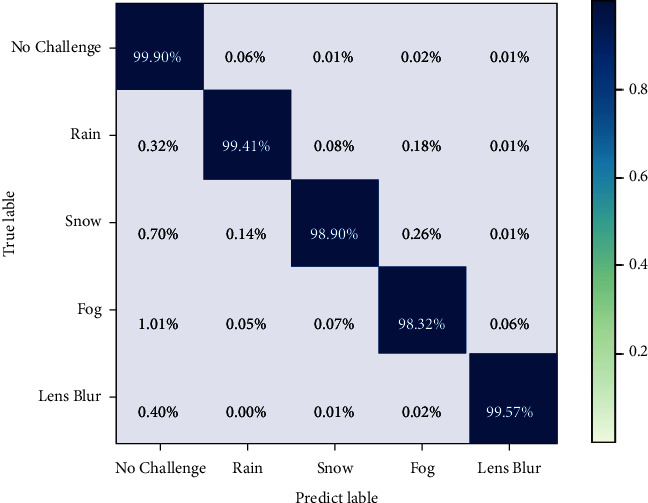
Performance of challenge classifier.

The performance of the Challenge Classifier has a crucial impact on our model. First, we use our enhanced dataset to test the performance of the classifier. Its classification accuracy reaches 99.32%, which is very satisfactory. Although our Challenge Classifier achieves such excellent performance, we still must consider whether the Challenge Classifier will classify the unchallenged image as the challenged one, which will lead to the degradation of image quality and affect the detection performance of subsequent traffic signs. As shown in the confusion matrix in Figure 9, our Challenge Classifier achieves a detection classification accuracy of 99.90% when detecting unchallenged images. Unchallenged images have an extremely low probability of being misclassified. Therefore, this hardly affects the performance of traffic sign detection. However, misclassification occurs in some challenges with low environmental complexity, such as light fog and snow, as they are very similar to normal weather.

We compare the improved YOLOv4 model with the current advanced detection models Faster-RCNN, SSD, RetinaNet, YOLOv3, and YOLOv4. The comparison results are listed in Table 1.

The proposed method achieves an mAP of 81.78% on the augmented TT-100K dataset. Compared with the classic YOLOv4, the mAP of our model is increased by 4.53%, with significant improvement. Compared with the single-stage detectors SSD, RetinaNet, YOLOv3, and YOLOv4-Tiny, the mAP of our model is 9.66, 9.94, 7.46, and 9.75% higher, respectively. Compared with the two-stage detector Faster-RCNN, our model is highly competitive in its mean average precision. Although the mAP of Faster-RCNN is slightly higher than our model, its extremely high number of floating point operations makes it difficult to apply in mobile devices.

To verify the performance of our model in different environments, we tested it under different complex environments, such as no challenge, rainy, snowy, foggy, and lens blur and compared it with the current mainstream model. We conducted multiple experiments, and the best results are shown in Table 1. The traffic sign precision under the no challenge environment is very high, mAP_NoChallenge_ reaching 87.19%, i.e., 3.27% higher than the classic YOLOv4, 8.08% higher than the YOLOv4-Tiny and compared with the one-stage detector SSD, RetinaNet and YOLOv3, mAP_NoChallenge_, it is increased by 8.48, 9.12, and 7.01%, respectively. Moreover, compared with the two-stage detector Faster-RCNN, the mAP_NoChallenge_ of our model is 1.67% lower; however, it is still more competitive. In addition, for the detection and recognition of traffic signs in complex environments, our model performs significantly better than other models in terms of detection. This is attributed to our pre-processing of the image before the detection of traffic signs, which enables the model to perform better in various complex environments.

Our model achieves the highest mAP in rainy, snowy, foggy, or lens blur conditions. Among these conditions, the model exhibits the best performance in traffic sign detection in blurred images, with mAP_LensBlur_ reaching 76.41%, which is 7.15% higher than the classic YOLOv4 and 3.36% higher than Faster-RCNN. In the foggy environment, the large amount of condensed water vapor in the air seriously affects the detection performance of the model. Therefore, the detection performance of each model is reduced under foggy conditions. Nevertheless, our model still achieves the highest performance for traffic sign detection under foggy conditions, with mAP_Fog_ of 72.50%, which is 6.91% higher than the classic YOLOv4 and 3.22% higher than Faster-RCNN. Furthermore, our improvement on the feature pyramid fuses deep features with shallow ones, thereby retaining more multiscale feature information, which allows us to extract more valuable information in complex environments and improve the model's performance.

In terms of real-time performance, our FPS is slightly lower than the original YOLOv4, while the speed advantage compared to other models remains evident. The decrease in FPS is mainly due to the addition of a classification denoizing model. However, owing to the modular design of the classification noise reduction model, we can consider whether to use this module as needed. Compared with the two-stage detector Faster-RCNN, our model achieves extremely high real-time performance. This recognition speed, which is three times higher than that of Faster-RCNN, provides a great opportunity for this model to be assembled on moving vehicles. As can be seen from Table 1, although YOLOv4-Tiny has a better real-time detection performance of 142 FPS, it has a significant decrease in accuracy. For traffic sign detection, 74 FPS already satisfies the real-time requirement. At this point, detection accuracy is more important, especially in extreme weather and for traffic sign detection and recognition of small objects. This improvement in accuracy can significantly improve driving safety. This is the reason why we choose YOLOv4 as the baseline model.

Although the addition of the Classify Denoizer module leads to a slight decrease in the overall model detection speed, we observe from the detection results that the number of accurately recognized traffic signs in the classified and denoized images is effectively improved. The speed of our model entirely meets real-time requirements in the field of autonomous driving and will not affect practical applications. After comparing the detection precision and speed with the mainstream model, our model is deemed superior.

#### 4.4.2. Ablation Study

To verify the performance improvement brought by different refinements to the proposed model, we conduct ablation studies to evaluate the effectiveness of the Classify Denoizer module, FT-FPN structure, EIOU loss, and Cluster-NMS.

Table 2 shows the impact of different improvements on the detection performance on YOLOv4. The mAP of the standard YOLOv4 is 77.25%. Considering that the enhanced Tsinghua-Tencent 100K dataset contains a large number of images in complex environments, we add the proposed image preprocessing module, the Classify Denoizer module, in front of YOLOv4. Owing to the modular design, this module can easily be added to or omitted from YOLOv4.

After adding the Classify Denoizer module, the model mAP reaches 79.15%. However, the recognition speed of the model decreases slightly, because our Classify Denoizer module first classifies the image and then performs corresponding noise reduction processing, thus affecting the overall recognition speed of the model. In addition, we use the improved FT-FPN structure to replace the original structure of YOLOv4 and do not add the Classify Denoizer. Thus, the mAP of the improved model improves from 77.25 to 79.36%. Furthermore, the addition of EIOU loss and Cluster-NMS has a certain improvement in the mean average precision of the model. Ablation experiments show that after combining the above-given modules, the performance of the model is greatly improved.

## 5. Conclusions

In this paper, we propose a multi-scale traffic sign detection method in complex environments based on improved YOLOv4. A Classify Denoizer module is added in front of the YOLOv4 model, which classifies the image by the types of noise and uses the Denoizing Block for noise reduction. We also improve the original feature pyramid to reduce the possible information loss during the generation of feature maps and enhance the information transfer between shallow and deep features. EIOU loss and Cluster-NMS are employed to further improve the detection performance. Our model exhibits a significant improvement in the precision of traffic signs in the rain, snow, fog, and lens blur and has better detection precision and real-time performance for multiscale traffic sign recognition. However, the detection performance of our model in other complex environments is still not satisfactory. Notably, the introduction of the Classify Denoizer module decreases the real-time performance of the model. In the future, we plan to improve the Classify Denoizer module to improve its detection speed and ability to deal with various complex environments.

## Figures and Tables

**Table 1:** Comparison of proposed and other methods.

Model	mAP_NoChallenge_	mAP_Rain_	mAP_Snow_	mAP_Fog_	mAP_LensBlur_	mAP	FPS
Faster-RCNN	88.86	70.51	71.07	69.28	73.05	82.59	24
SSD	78.71	61.54	63.56	60.97	64.58	72.12	51
RetinaNet	78.07	59.55	60.91	63.89	64.76	71.84	53
YOLOv3	80.18	63.94	63.83	62.81	66.25	74.32	58
YOLOv4-tiny	79.11	61.57	61.45	60.32	63.59	72.03	142
YOLOv4	83.92	66.62	67.14	65.59	69.26	77.25	86
Ours	87.19	73.22	74.02	72.50	76.41	81.78	74

**Table 2:** Ablation experiment results.

Method	mAP (%)
YOLOv4	77.25
YOLOv4 + classify denoizer	79.15
YOLOv4 + FT-FPN	79.36
Proposed model	81.78

## Data Availability

The dataset studied in this paper can be obtained from Tsinghua-Tencent 100K official website.
